# The length of stay in the post-anaesthesia care unit correlates with pain intensity, nausea and vomiting on arrival

**DOI:** 10.1186/s13741-014-0010-8

**Published:** 2014-11-26

**Authors:** Michael T Ganter, Stephan Blumenthal, Seraina Dübendorfer, Simone Brunnschweiler, Tim Hofer, Richard Klaghofer, Andreas Zollinger, Christoph K Hofer

**Affiliations:** Institute of Anaesthesiology and Pain Medicine, Kantonsspital Winterthur, Brauerstrasse 15, 8401 Winterthur, Switzerland; Institute of Anaesthesiology and Intensive Care Medicine, Triemli City Hospital Zurich, Birmensdorferstrasse 497, 8063 Zurich, Switzerland; Department of Psychiatry and Psychotherapy, Statistics, University Hospital Zurich, Raemistrasse 100, 8006 Zurich, Switzerland

**Keywords:** Post-anaesthesia care unit (PACU), Postoperative pain, Postoperative nausea, Postoperative vomiting, PONV

## Abstract

**Background:**

The benefit of the post-anaesthesia care unit (PACU) with respect to an early detection of postoperative complications is beyond dispute. From a patient perspective, prevention and optimal management of pain, nausea and vomiting (PONV) are also of utmost importance. The aims of the study were therefore to prospectively measure pain and PONV on arrival to the PACU and before discharge and to determine the relationship of pain and PONV to the length of stay in the PACU.

**Methods:**

Postoperative pain was assessed over 30 months using a numeric rating scale on admittance to the PACU and before discharge; in addition, PONV was recorded. Statistical analysis was done considering gender, age, American Society of Anesthesiologists (ASA) classification, surgical speciality, anaesthesia technique, duration of anaesthesia, intensity of nursing and length of stay.

**Results:**

Data of 12,179 patients were available for analysis. The average length of stay in the PACU was 5.7 ± 5.9 h, whereas regular PACU patients stayed for 3.2 ± 1.9 h and more complex IMC patients stayed for 15.1 ± 6.0 h. On admittance, 27% of patients were in pain and the number decreased to 13% before discharge; 3% experienced PONV. Risk factors for increased pain determined by multivariate analysis were female gender; higher ASA classification; general, cardiac and orthopaedic surgery and prolonged case duration. In more complex IMC patients, pain scores were higher on arrival but dropped to similar levels before discharge compared to regular PACU patients. Female gender and postoperative pain were risk factors for postoperative vomiting. Pain and PONV on arrival correlated with length of stay in the PACU. Pain- or PONV-free patients stayed almost half of the time in the PACU compared to patients with severe pain or vomiting on arrival.

**Conclusions:**

The majority of PACU patients had good pain control, both on admittance and before discharge, and the overall incidence of PONV was low. Managing patients in the PACU could achieve a significant reduction of pain and PONV. The level of pain and presence of PONV on admittance to the PACU correlate with and act as predictors for increased length of PACU stay.

## Background

Today, post-anaesthesia care units (PACUs) are a standard and integral part of daily anaesthesia practice in most developed countries. Their main purpose is to safely recover patients from surgical and interventional procedures with concomitant anaesthesia. Through structured and tight clinical observation combined with continuous patient monitoring, discomfort and complications can be identified and treated early, thereby reducing adverse outcomes and increasing efficacy [1]. Several practice guidelines for post-anaesthesia care are available and have been recently updated, such as those from the European Board of Anaesthesiology and the American Society of Anesthesiologists [2,3].

The benefit of a PACU service with respect to early detection of postoperative complications is beyond dispute. From a patient perspective, however, prevention and optimal management of pain, nausea and vomiting are of utmost importance as well. Therefore, the degree of pain, quantity of pain relief over time and the presence of postoperative nausea and vomiting (PONV) are being used as popular quality indicators for anaesthesia and postoperative care in PACU audits [2,4].

The *first* aim of the present study was to prospectively measure pain and PONV at two defined time points: on arrival to our PACU and before discharge to the ward. As a quality management project, we wanted to specifically monitor these indicators and to compare our results to published reports. The *second* aim of the study was to determine if pain and PONV on arrival correlate with the length of stay in our PACU. It is important to early identify patients at risk for unplanned, prolonged PACU stay for optimal perioperative efficiency [5]. If the PACU becomes congested by these patients, the outflow of patients from the PACU to the ward is stopped and the unit cannot receive any further patients from the operating room (OR).

## Methods

After approval from the local ethic committee (Kantonale Ethik Kommission, 8090 Zurich, Switzerland: KEK-StV-Nr. 47/12), which waived the requirement for informed consent, the anaesthesia quality management project was established. Data were prospectively collected in the PACU of the Triemli City Hospital Zurich, Switzerland during a time period of 30 months.

### Postoperative patient flow and care in our hospital

Depending on patient- and procedure-related factors, patients are either being recovered from surgical and interventional procedures with concomitant anaesthesia in the PACU or are being sent directly to the ward (inpatients to the regular, surgical ward; outpatients to the ambulatory holding area). Except for the ICU, our PACU represents the only unit where continuous patient monitoring and a higher level of nursing care can be provided after anaesthesia. Our PACU, open 24/7, has ten beds with continuous full patient monitoring. The PACU staffing consists of a nurse to bed ratio of 1:3 on an average per day shift on a week day; the surgical ward has a ratio of 1:6 and no continuous patient monitoring available.

Patients are being admitted to the PACU when patient- or procedure-related factors require specific PACU care, i.e. co-morbidities, American Society of Anesthesiologists (ASA) classification ≥3, prolonged anaesthesia and major surgery, general anaesthesia with neuromuscular blockade, starting and titrating opioid or local anaesthetic pain pumps or mandatory prolonged postoperative observation. Patients stayed overnight, if necessary.

### Baseline data and patient categories

Patient characteristics (gender, age, ASA classification) and case-related data (surgical speciality, type and duration of anaesthesia, length of stay in the PACU) of all PACU patients were recorded. To measure nursing workload and complexity, NEMS (nine equivalents of nursing manpower use score [6]) was calculated for every patient. Thereby, patients were categorized into two groups: *regular* PACU patients (NEMS ≤15), and *more complex* intermediate care patients (IMC; NEMS >15).

### Postoperative pain, nausea and vomiting

On admittance to the PACU and before discharge to the regular ward, patients were asked for pain, nausea and vomiting. Level of pain was assessed using the numeric rating scale (NRS, graded from zero to ten), and patients were asked to express the intensity of their actual pain score in numbers [7].

Pain management was done according to institutional standards using a multimodal approach [8,9]. Depending on the underlying and concomitant diseases, analgesia was done with paracetamol, non-steroidal anti-inflammatory drugs (NSAID’s), metamizol or a combination thereof. Next, when NRS pain intensity was greater than four, intravenous opioids (morphine, fentanyl or meperidine) were added. For major abdominal, thoracic and orthopaedic surgery, combined anaesthesia was performed and continued throughout the postoperative period whenever feasible. If there were contraindications for regional anaesthesia techniques, IV patient-controlled analgesia (PCA) pumps with opioids were initiated in the PACU and continued postoperatively.

Preoperatively, a systematic risk assessment for PONV was done for all patients. Regional anaesthesia was favoured for patients at risk for PONV whenever possible. For patient-related risk factors, we used the Apfel simplified risk score [10]. Together with procedure-related risk factors (e.g. type and duration of surgery, postoperative opioids), anaesthesia was planned together with a multimodal approach to minimize PONV [11]. For example, all patients with a risk score of two received total intravenous anaesthesia combined with a 5-HT3 antagonist (IV granisetron). For higher risk (i.e. Apfel score ≥3), additional IV dexamethasone was given. For PONV treatment in the PACU, gradual therapy was administered using metoclopramid, granisetron, dexamethasone and droperidol [3]. In case of persistent, massive nausea and protracted vomiting, low dose propofol was given [12].

### Statistics

Data were analyzed using SPSS for Windows, Release 12.0.0 (SPSS Inc., Chicago, IL) and Statview 5.01 Software (SAS Institute Inc., Cary, USA). Statistical analysis was performed for the total patient collective and the following subgroups: gender, age, ASA classification, surgical specialities, anaesthetic technique, duration of anaesthesia and intensity of nursing care. *χ*^2^-test was used to compare pain intensity and incidence of PONV on admittance to the PACU and before discharge as well as to compare subgroups. To calculate predictors for length of PACU stay and postoperative pain, a linear multiple regression analysis (displaying ß-weight and significance levels) was used. Additionally, logistic regression analysis (displaying odds ratio and 95% confidence intervals) was done to calculate predictors for postoperative nausea and vomiting. Data are given as mean value ± standard deviation (SD). A *p* value <0.05 was considered statistically significant.

## Results

A total of 16,309 patients were cared for in the PACU during a time period of 30 months. This collective represents 41% of all patients that underwent anaesthesia in this time period. The data of 12,179 patients were available for statistical analysis. Sets of data (4,130) had to be excluded because our questions related to pain and PONV could not be answered reliably. These patients had language difficulties or suffered from concomitant neurologic/psychiatric diseases. Patient characteristics and case related data are presented in Table 1.

**Table 1:** **Patient characteristics and case-related data of 12,179 PACU patients**

		**Numbers,** ***n*** **(%)**		**Length of stay, hours**		
				**All patients**	**Regular PACU**	**IMC**
Gender	Women	5,866	(48)	6.1 ± 5.9	3.4 ± 2.1	14.7 ± 6.2*
	Men	6,313	(52)	5.4 ± 5.8**	2.9 ± 1.8**	15.6 ± 5.7*^,^**
Age	<40 years	1,312	(11)	4.4 ± 4.8	2.9 ± 1.8	14.4 ± 6.7*
	40–80 years	8,009	(66)	5.7 ± 6.0**	3.1 ± 2.0**	15.8 ± 5.7*^,^**
	>80 years	2,858	(24)	6.4 ± 6.1**	3.3 ± 2.1**	14.0 ± 6.4*
ASA classification	ASA I	1,949	(16)	3.7 ± 3.9	2.8 ± 1.6	14.3 ± 6.5*
	ASA II	5,480	(45)	5.4 ± 5.6**	3.1 ± 2.0**	15.3 ± 5.8*^,^**
	ASA III	4,628	(38)	6.9 ± 6.5**	3.3 ± 2.2**	15.1 ± 6.1*
	ASA IV	122	(1)	9.8 ± 8.4**	4.4 ± 3.3**	15.7 ± 8.3*
Surgical specialities	General surgery	7,237	(59)	6.4 ± 6.3	3.4 ± 2.1	14.6 ± 6.3*
	Cardiac surgery	667	(5)	6.8 ± 6.9	2.8 ± 1.6**	17.3 ± 4.1*^,^**
	Orthopaedic surgery	675	(6)	7.1 ± 5.4**	4.8 ± 1.9**	16.1 ± 4.9*^,^**
	Spine surgery	603	(6)	6.4 ± 5.8	3.5 ± 2.1	15.1 ± 4.3*
	Urology	1,584	(13)	5.0 ± 4.6**	2.7 ± 1.6**	16.9 ± 4.9*^,^**
	Ophthalmology	1,267	(10)	2.3 ± 1.9**	2.1 ± 0.9**	14.2 ± 4.5*^,^**
	ENT surgery	146	(1)	3.8 ± 4.2**	2.6 ± 1.5**	16.7 ± 3.5*
Anaesthetic techniques	MAC	436	(3)	4.7 ± 5.7	2.7 ± 1.5**	16.9 ± 5.9*^,^**
	Peripheral RA	76	(1)	5.8 ± 6.9	4.0 ± 2.1	12.2 ± 5.7*^,^**
	Central neuraxial RA	1,048	(9)	4.1 ± 4.3**	3.0 ± 1.8	15.6 ± 5.7*
	General anaesthesia	8,979	(74)	5.3 ± 5.7	3.0 ± 1.9	15.1 ± 6.0*
	Combined anaesthesia	1,640	(13)	8.8 ± 6.8**	4.3 ± 2.3**	15.0 ± 5.9*
Duration of anaesthesia	<60 min	196	(4)	5.0 ± 6.1	2.6 ± 1.5	16.2 ± 6.7*
	60–180 min	7,888	(63)	4.0 ± 4.1**	2.9 ± 1.7	14.1 ± 6.6*
	>180 min	4,095	(33)	8.8 ± 7.1**	3.9 ± 2.4**	15.5 ± 5.7*
Intensity of nursing care	Regular PACU/IMC	9,603/2,576	(78/22)	5.7 ± 5.9	3.2 ± 2.3	15.1 ± 6.0*

*ASA classification* American Society of Anesthesiologists’ classification, *ENT* ear-nose-throat, *IMC* intermediate care (more complex, intermediate care patients, NEMS >15), *MAC* monitored anaesthesia care, *PACU* post-anaesthesia care unit (regular post-anaesthesia care unit patients, NEMS ≤15), *RA* regional anaesthesia.

*p <0.05 (comparison PACU-IMC), **p <0.05 (age: comparison with age <40 years; ASA classification: comparison with ASA I; surgical specialities: comparison with general surgery; anaesthetic technique: comparison with general anaesthesia; duration of anaesthesia: comparison with duration <60 min).

**Figure 1:**
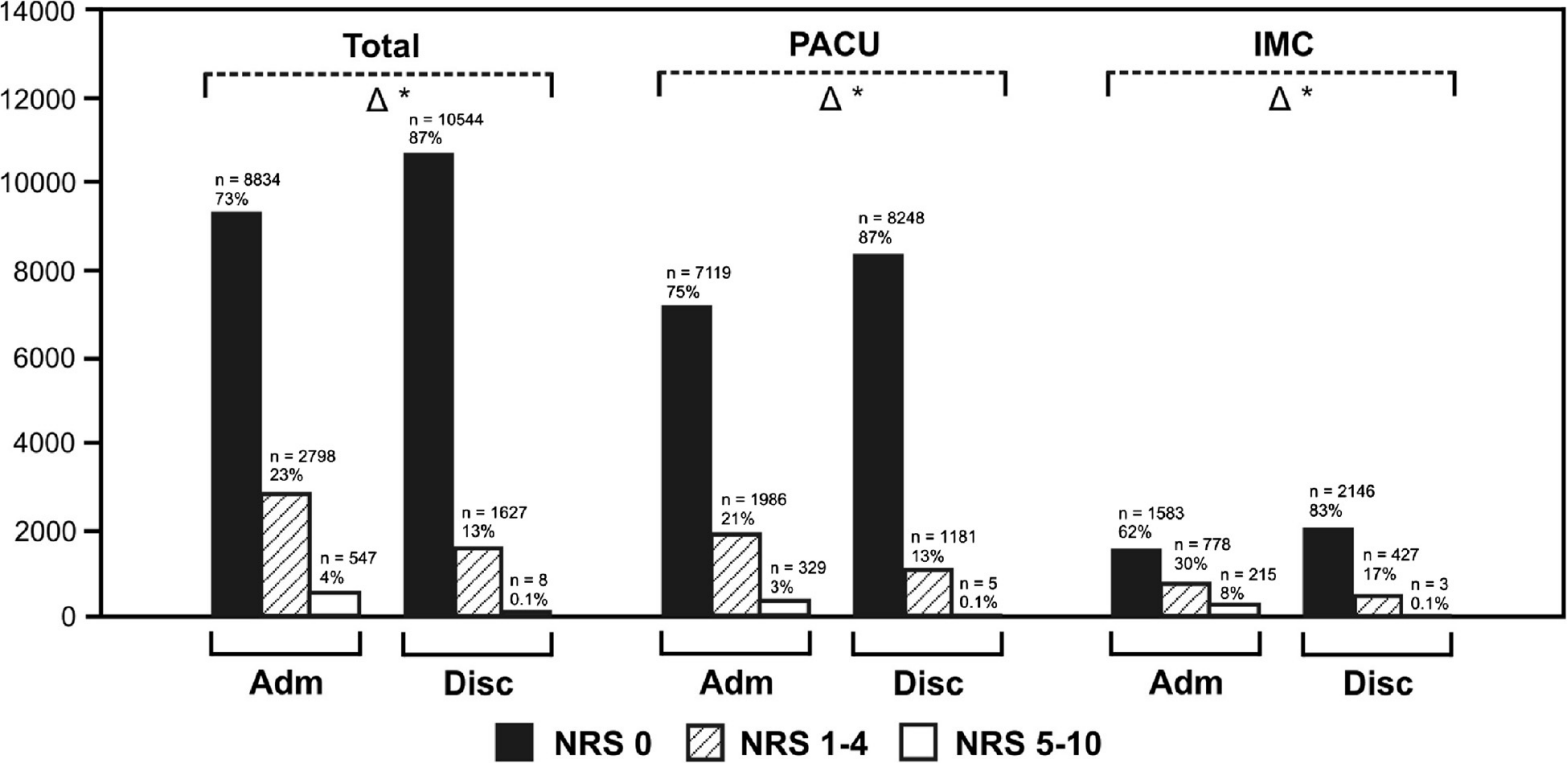
**Incidence and severity of pain assessed using a NRS on admittance to the post-anaesthesia care unit and before discharge to the regular ward.** *PACU* = regular post-anaesthesia care unit patients (NEMS ≤15), *IMC* = more complex, intermediate care patients (NEMS >15), *NRS* = numeric rating scale. *Black bars*: patients with NRS = 0, *striped bars*: patients with NRS 1–4, *white bars*: patients with NRS 5–10. Difference (*triangle*) between admittance (*Adm*) and discharge (*Disc*), *single asterisks*: *p* <0.05.

On admittance to the PACU, 73% of patients were free of pain (NRS 0), 23% of patients had minor pain (NRS 1–4) and 4% of patients suffered from severe pain (NRS 5–10). Before discharge, 87% of patients were pain free, 13% of patients had minor pain and only 0.1% patients suffered from major pain. There was a significant difference in the presence of pain (NRS 1–10) on admission between regular PACU (24%) and more complex IMC (38%) patients (Figure 1). Incidence of postoperative pain for different subgroups is presented in Table 2. Risk factors for increased postoperative pain (on admittance) determined by multivariate analysis were female gender; higher ASA classification; general, cardiac and orthopaedic surgery and prolonged case duration. Furthermore, the performance of general anaesthesia without the combination of regional anaesthesia was an independent risk factor. By contrast, old age, ophthalmologic procedures or ENT surgery were predictive for lower pain scores (Table 3).

**Table 2:** **Incidence of postoperative pain**

		**Pain on admittance,** ***n*** **(%)**			**Pain before discharge,** ***n*** **(%)**		
		**NRS 0**	**NRS 1–4**	**NRS 5–10**	**NRS 0**	**NRS 1–4**	**NRS 5–10**
Gender	Women	4,085 (69)	1,528 (26)	279 (5)	5,029 (85)	857 (15)	6 (0)*
	Men	4,775 (76)	1,268 (20)	270 (4)**	5,539 (88)	770 (12)	4 (0)*^,^**
Age	<40 years	801 (61)	416 (32)	95 (7)	1,030 (78)	281 (21)	1 (1)*
	40–80 years	5,789 (72)	1,850 (23)	370 (5)**	6,915 (86)	1,087 (14)	7 (0)*
	>80 years	2,244 (78)	530 (19)	84 (3)**	2,597 (91)	259 (9)	2 (0)*^,^**
ASA classification	ASA I	1,370 (69)	510 (26)	98 (5)	1,644 (83)	332 (17)	2 (0)*
	ASA II	4,008 (73)	1,246 (23)	253 (4)**	4,763 (86)	768 (14)	3 (0)*^,^**
	ASA III	3,375 (74)	1,009 (22)	194 (4)**	4,066 (89)	508 (11)	4 (0)*^,^**
	ASA IV	81 (70)	31 (27)	4 (3)	96 (83)	19 (16)	1 (1)*
Surgical specialities	General surgery	4,872 (67)	1,961 (27)	404 (6)	6,081 (84)	1,151 (16)	5 (0)*
	Cardiac surgery	561 (84)	96 (14)	10 (2)	620 (93)	47 (7)	0 (0)*^,^**
	Orthopaedic surgery	433 (64)	194 (29)	48 (7)**	548 (81)	127 (19)	0 (0)*^,^**
	Spine surgery	403 (67)	155 (26)	45 (7)	499 (83)	102 (17)	2 (0)*
	Urology	1,357 (86)	198 (12)	29 (2)**	1,496 (94)	88 (6)	3 (0)*^,^**
	Ophthalmology	1,102 (87)	160 (13)	5 (0)**	1,188 (94)	79 (6)	0 (0)*^,^**
	ENT surgery	120 (82)	25 (17)	1 (1)**	131 (90)	15 (10)	0 (0)*^,^**
Anaesthetic technique	MAC	391 (89)	42 (10)	3(1)**	404 (93)	32 (7)	0 (0)*^,^**
	Peripheral RA	64 (84)	12 (16)	0 (0)**	71 (93)	5 (7)	0 (0)*
	Central neuraxial RA	973 (92)	62 (7)	13 (1)**	966 (92)	82 (8)	0 (0)*^,^**
	General anaesthesia	6,220 (69)	2,303 (26)	456 (5)	7,692 (85)	1,280 (15)	7 (0)
	Combined anaesthesia	1,187 (73)	377 (23)	68 (4)	1,411 (86)	228 (14)	1 (0)*
Duration of anaesthesia	<60 min	162 (83)	30 (15)	4 (2)	176 (90)	20 (10)	0 (0)*
	60–180 min	6,042 (77)	1,610 (20)	236 (3)	6,973 (88)	909 (12)	6 (0)*
	>180 min	2,630 (64)	1,156 (28)	309 (8)**	3,393 (83)	698 (17)	4 (0)*^,^**
Intensity of nursing care	PACU	7,288 (66)	1,986 (21)	329 (5)	8,417 (88)	1,181 (12)	5 (0)*
	IMC	1,583 (62)	778 (30)	215 (8)**	2,146 (83)	427 (17)	3 (0)*^,^**

*ASA classification* American Society of Anesthesiologists’ classification, *ENT* ear-nose-throat, *IMC* intermediate care (more complex, intermediate care patients, NEMS >15), *MAC* monitored anaesthesia care, *NRS* numeric rating scale, *PACU* post-anaesthesia care unit (regular post-anaesthesia care unit patients, NEMS ≤15), *RA* regional anaesthesia.

*p <0.05 (comparison admittance-discharge), **p <0.05 (gender: comparison with women; age: comparison with age <40 years; ASA classification: comparison with ASA I; surgical specialities: comparison with general surgery; anaesthetic technique: comparison with general anaesthesia; duration of anaesthesia: comparison with duration <60 min; intensity of nursing: comparison with PACU).

**Table 3:** **Multivariate analysis**

	**Length of stay**	**Postoperative pain**	**Postoperative nausea**		**Postoperative vomiting**	
**Independent variables**	**ß**	**ß**	**OR**	**95% CI**	**OR**	**95% CI**
Gender	0.008	0.055**	2.455**	1.783/3.382	3.194**	1.755/5.816
Age	0.036**	−0.148**	0.988*	0.979/0.998	0.992	0.974/1.009
ASA classification	0.159**	0.062**	1.101	0.859/1.410	0.915	0.593/1.414
Surgical specialities						
General surgery	−0.047	0.074*	0.511	0.232/1.126	0.584	0.138/2.467
Cardiac surgery	0.058**	0.062**	0.411	0.128/1.325	0.781	0.091/6.678
Orthopaedic surgery	−0.021	0.099**	0.583	0.223/1.525	0.853	0.162/4.484
Spine surgery	0.058**	0.014	0.450	0.168/1.206	0.716	0.133/3.853
Urology	−0.073**	−0.015	0.015	0.979/0.998	0.530	0.093/3.036
Ophthalmology	−0.126**	−0.073**	0.299*	0.107/0.834	0.250	0.034/1.856
ENT surgery	−0.071**	−0.036**	0.689	0.170/2.792	n.a.	n.a.
Anaesthetic technique						
MAC	−0.055**	0.028*	3.825*	1.172/12.485	0.813	0.090/7.330
RA	−0.001	−0.046**	2.675*	1.047/6.830	0.422	0.049/3.653
General anaesthesia	0.041**	0.104**	1.365	0.708/2.633	0.607	0.251/1.468
Combined anaesthesia	0.031*	0.010	1.101	0.514/2.359	0.647	0.226/1.851
Duration of anaesthesia	0.432**	0.144**	1.003**	1.001/1.005	1.003	1.000/1.006
Postoperative pain	0.051**	n.a.	1.161**	1.084/1.244	1.224*	1.099/1.363
Postoperative nausea/vomiting	0.025**	n.a.	n.a.	n.a.	n.a.	n.a.

To calculate predictors for length of PACU stay and postoperative pain, linear multiple regression analysis (displaying ß-weight = standardized coefficient) was used; to calculate predictors for postoperative nausea and vomiting, logistic regression analysis (displaying OR = odds ratio, 95% CI = 95% confidence interval) was used.

*ASA classification* American Society of Anesthesiologists’ classification, *ENT* ear-nose-throat, *MAC* monitored anaesthesia care, *n.a.* not applicable, *RA* regional anaesthesia (peripheral and central neuraxial).

*p <0.05, **p <0.001.

**Figure 2:**
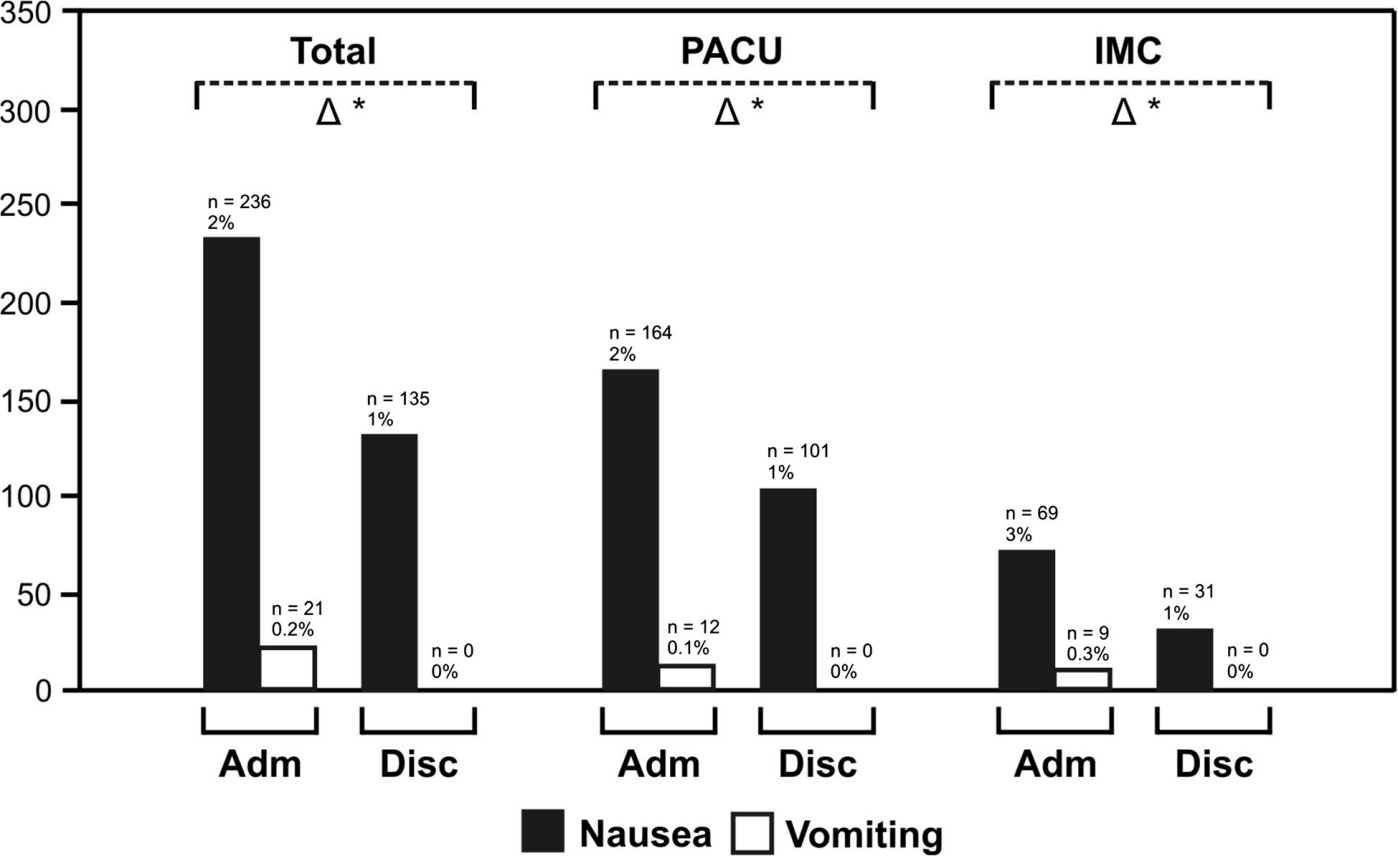
**Incidence of postoperative nausea and vomiting on admittance to the post-anaesthesia care unit and before discharge to the regular ward.** *PACU* = regular post-anaesthesia care unit patients (NEMS ≤15), *IMC* = more complex, intermediate care patients (NEMS >15). *Black bars*: patients with postoperative nausea, *white bars*: patients with postoperative vomiting. Difference (*triangle*) between admittance (*Adm*) and discharge (*Disc*), *single asterisks*: *p* <0.05.

PONV was observed in 257 patients on admittance to the PACU, whereas 21 patients were vomiting. Until discharge to the ward, 224 of these 257 patients were free of symptoms and 103 patients (0.8%) developed new nausea (data not shown). Therefore at the time of discharge, a total of 135 patients had nausea but none suffered from vomiting (Figure 2). A total of 360 patients (3%) had PONV during the recovery period. Subgroup analysis showed a significant reduction of postoperative nausea and vomiting during the PACU stay for all patient groups except for those undergoing spine surgery and patients with higher ASA classification (Table 4). Patients with major pain levels (NRS 5–10) on admittance had a higher incidence of postoperative nausea (5.3%) and vomiting (1.3%) compared to patients with lower pain levels (NRS 0–4; nausea 2.2%, vomiting 0.2%, *p* <0.05). Risk factors for nausea in the multiple linear regression analysis were female gender, monitored anaesthesia care and performance of regional anaesthesia. By contrast, reduced nausea occurred in older patients and a patient undergoing ophthalmologic procedures. The only independent risk factors for postoperative vomiting were female gender and postoperative pain (Table 3).

**Table 4:** **Incidence of postoperative nausea and vomiting (PONV)**

		**Nausea,** ***n*** **(%)**		**Vomiting,** ***n*** **(%)**	
		**On admittance**	**Before discharge**	**On admittance**	**Before discharge**
Gender	Women	163 (3)	103 (2)*	15 (0.3)	0 (0)*
	Men	73 (1)**	32 (1)*	6 (0.2)	0 (0)*
Age	<40 years	38 (3)	17 (1)*	4 (0.4)	0 (0)*
	40–80 years	134 (2)**	87 (1)*	16 (0.4)	0 (0)*
	>80 years	64 (2)**	(1)*	1 (0)	0 (0)
ASA classification	ASA I	33 (2)	21 (1)*	2 (0)	0 (0)
	ASA II	115 (2)	65 (1)*	15 (1) **	0 (0)*
	ASA III	88 (2)	48 (1)*	4 (0)	0 (0)
	ASA IV	0 (0)	1 (1)*	0 (0)**	0 (0)
Surgical specialities	General surgery	146 (2)	88 (1)*	13 (0)	0 (0)*
	Cardiac surgery	12 (3)	3 (1)*	0 (0)	0 (0)
	Orthopaedic surgery	18 (3)	5 (1)*	2 (1)	0 (0)*
	Spine surgery	12 (2)	17 (3)	5 (1)	0 (0)*
	Urology	24 (1)**	15 (1)*	0 (0)	0 (0)
	Ophthalmology	11 (1)**	5 (0)*	0 (0)	0 (0)
	ENT surgery	3 (2)	0 (0)*	0 (0)	0 (0)
Anaesthetic technique	MAC	12 (3)	6 (1)*	0 (0)	0 (0)
	Peripheral RA	1 (1)	0 (0)*	0 (0)	0 (0)
	Central neuraxial RA	12 (2)	6 (1)*	1 (0)	0 (0)
	General anaesthesia	178 (2)	104 (1)*	19 (0)	0 (0)*
	Combined anaesthesia	33 (2)	19 (1)*	1 (0)	0 (0)
Duration of anaesthesia	<60 min	6 (3)	3 (2)*	1 (1)	0 (0)
	60–180 min	120 (4)	77 (1)*	7 (0)	0 (0)
	>180 min	110 (3)	55 (1)*	13 (0)	0 (0)*
Intensity of nursing care	PACU	164 (2)	101 (1)*	12 (0.1)	0 (0)*
	IMC	69 (3)**	31 (1)*	9 (0.3)	0 (0)*

*ASA classification* American Society of Anesthesiologists’ classification, *ENT* ear-nose-throat, *IMC* intermediate care (more complex intermediate care patients, NEMS >15), *MAC* monitored anaesthesia care, *NRS* numeric rating scale, *PACU* post-anaesthesia care unit (regular post-anaesthesia care unit patients NEMS ≤15), *RA* = regional anaesthesia.

*p <0.05 (comparison admittance-discharge), **p <0.05 (gender: comparison with women; age: comparison with age <40 years; ASA classification: comparison with ASA I; surgical specialities: comparison with general surgery; anaesthetic technique: comparison with general anaesthesia; duration of anaesthesia: comparison with duration <60 min; intensity of nursing: comparison with PACU).

**Figure 3:**
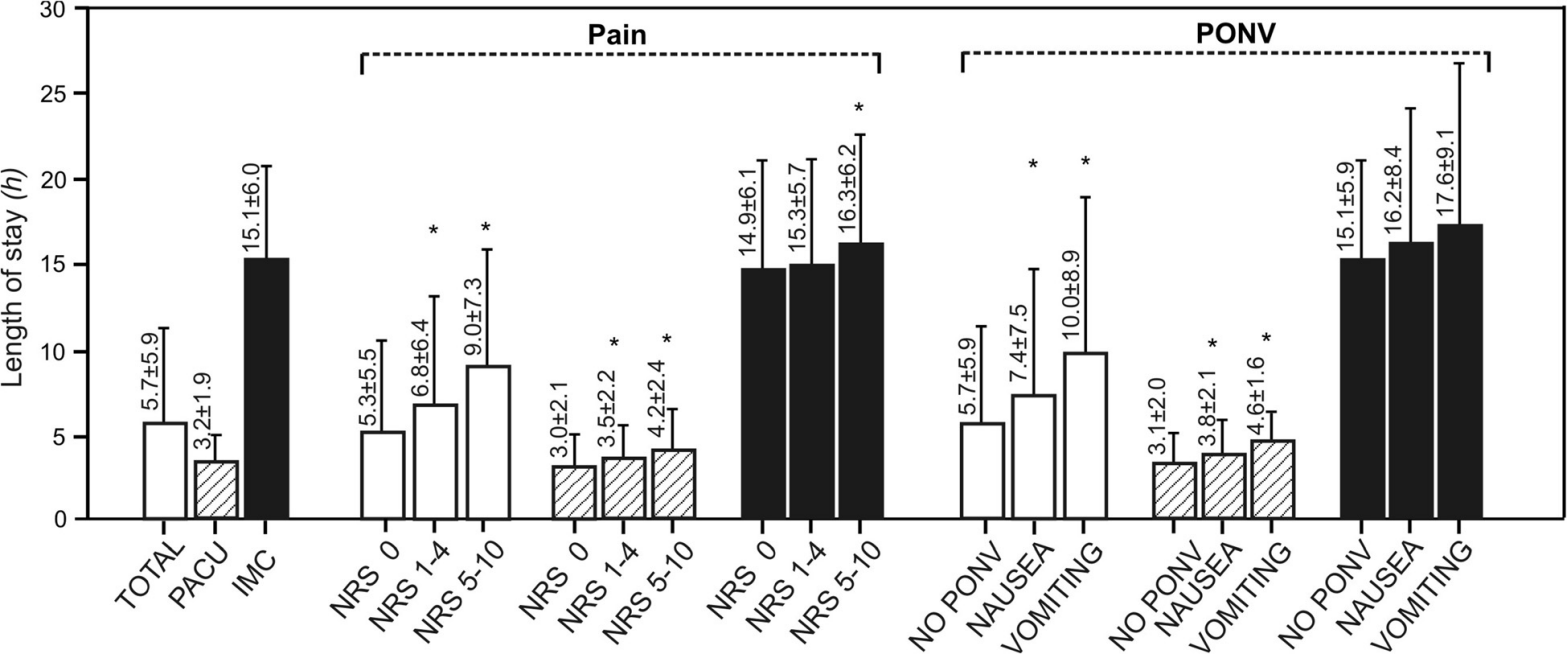
**Length of stay in the post-anaesthesia care unit in relation to pain and PONV on admittance.** *PACU* = regular post-anaesthesia care unit patients (NEMS ≤15), *IMC* = more complex, intermediate care patients (NEMS >15), *NRS* = numeric rating scale, *PONV* = postoperative nausea and vomiting. *White bars*: all patients (total), *stripped bars*: regular post-anaesthesia care unit patients (PACU, NEMS ≤15), *black bars*: more complex, intermediate care patients (IMC, NEMS >15), *single asterisks*: *p* <0.05 comparing to NRS 0 or NO PONV.

Duration of PACU stay was longer in a woman compared to a man and PACU stay became longer with increasing age, ASA classification and anaesthesia time (Table 1). Levels of pain and presence of PONV on admittance correlated with the length of stay: for example, patients with no pain or no PONV stayed for 5.3 ± 5.5 or 5.7 ± 5.9 h, whereas patients with severe pain (NRS 5–10) or nausea/vomiting stayed significantly longer (9.0 ± 7.3 or 7.4 ± 7.5/10.0 ± 8.9 h; Figure 3). Risk factors for prolonged stay, as determined by multiple regression analysis, are shown in Table 3.

## Discussion

In the present PACU quality audit, the majority of the studied 12,179 patients had good pain control (73% and 87% of patients were pain free on arrival and on discharge to the ward, respectively) and the incidence of PONV was 3% overall. In more complex IMC patients, pain scores were higher on arrival but dropped to similar levels on discharge compared to regular PACU patients. The levels of pain and presence of PONV on arrival correlated with the length of stay in the PACU. Thereby, pain- or PONV-free patients stayed almost half of the time in the PACU compared to patients with severe pain or vomiting on arrival.

The incidence of early postoperative pain in our study (Figure 1, Table 2) is only half or even less of commonly cited postoperative pain levels in the literature [13-15]. Several reasons may explain this difference: our patients were asked about their postoperative pain during their PACU stay with a simple numeric rating scale only, whereas the other studies performed more complex telephone or written surveys several weeks and months after the surgical procedures and asked for global postoperative pain experience. The results are therefore not comparable since two different things were measured.

Prompt and appropriate management of pain initiated in a PACU environment and continued on a regular ward may have a positive impact on patient satisfaction and perception of pain according to results of studies on the implementation of PACU-based pain services [16,17]. Our data are in accordance with these recommendations and show that pain relief was effective in our PACU resulting in a significant reduction of postoperative pain from admittance to discharge (Figure 1). Multivariate analysis revealed a variety of risk factors for increased postoperative pain (Table 3). Our data agree with previously published large trials, where younger age and type of surgery (e.g. major general, orthopaedic and cardiac surgery under general anaesthesia) were predictors for postoperative pain [13,18,19]. Concerning female gender as a predictor for increased postoperative pain, studies report conflicting results, however [19]. Patients at risk for increased postoperative pain may have a clear benefit from postoperative care in a PACU. On the other hand, patients undergoing surgery under regional anaesthesia may initially present in a pain free state. Nevertheless, PACU pain management can still be appropriate for these patients, e.g. to adjust continuous regional anaesthesia or to initiate and titrate systemic intravenous analgesia.

The incidence of PONV revealed in this study was much lower than the 25%–30% found in the literature (Figure 2, Table 4) [11]. This might be explained by the fact that preoperatively a systematic risk assessment was done for PONV in all patients and regional anaesthesia favoured whenever possible. According to the present study, the care in our PACU was able to significantly reduce postoperative nausea. A reduction of approximately 50% was observed in the total patient population as well as in the majority of the subgroups. Postoperative nausea was completely controlled during the PACU stay for all patients. The fact that women are especially prone to PONV has been demonstrated repeatedly [10] and is again supported by our data. Female patients obviously benefit from a post-anaesthesia care service.

An increased incidence of PONV was observed in patients with higher pain scores on PACU admittance. This finding may be explained by a higher use of opioids in order to control postoperative pain. Only recently, a study evaluating the relationship between postoperative opioid administration and PONV has been published and a logarithmic dose-effect relationship could be established [20]. In the present study, we did not record the individual doses and dosing intervals of opioids. However, we could show that postoperative pain is an independent predictor for PONV (Table 3). Based on these findings, profound intraoperative and early postoperative analgesia should be promoted to minimise deliberate use of postoperative opioids. This goal may be achieved by an increased use of multimodal analgesia techniques, including regional anaesthesia or combined anaesthetic techniques [21,22].

A limitation of the present investigation is the heterogeneity of the studied patient population with regard to the surgical specialities and the intraoperative anaesthetic technique. On the other side, by representing daily clinical reality, this heterogeneity could also be interpreted as strength. Additionally, our pain scores were not compared to preoperative values and both pain and PONV were just measured in the PACU period. To get more detailed information and to increase validity, it would be interesting for future studies to compare pain levels to baseline levels before and to add a follow-up on pain and PONV experience several weeks after their PACU stay.

## Conclusions

We conclude that the majority of PACU patients had good pain control, both on admittance and before discharge and that the overall incidence of PONV was low. Managing patients in the PACU could achieve a significant reduction of pain and PONV. The level of pain and presence of PONV on admittance to the PACU correlate with and act as predictors for increased length of PACU stay.

## Abbreviations

IMC: intermediate care
NEMS: nine equivalents of nursing manpower use score
NRS: numeric rating scale
OR: operating room
PACU: post-anaesthesia care unit
PCA: patient-controlled analgesia
PONV: postoperative nausea and vomiting
